# A Short Review on the Role of the Metal-Graphene Hybrid Nanostructure in Promoting the Localized Surface Plasmon Resonance Sensor Performance

**DOI:** 10.3390/s19040862

**Published:** 2019-02-19

**Authors:** Raed Alharbi, Mehrdad Irannejad, Mustafa Yavuz

**Affiliations:** 1 Mechanical Engineering Department, Taibah University, Madina P.O. Box. 344, Saudi Arabia; 2 Mechanical and Mechatronics Engineering Department, University of Waterloo, Waterloo, ON N2L 3G1, Canada; pm07mi@gmail.com (M.I.); myavuz@uwaterloo.ca (M.Y.); 3 OZ optics, Ltd., Ottawa, ON K0A 1L0, Canada

**Keywords:** localized plasmons, sensor, graphene, metal, hybrid, sensitivity, figure of merit

## Abstract

Localized Surface Plasmon Resonance (LSPR) sensors have potential applications in essential and important areas such as bio-sensor technology, especially in medical applications and gas sensors in environmental monitoring applications. Figure of Merit (FOM) and Sensitivity (S) measurements are two ways to assess the performance of an LSPR sensor. However, LSPR sensors suffer low FOM compared to the conventional Surface Plasmon Resonance (SPR) sensor due to high losses resulting from radiative damping of LSPs waves. Different methodologies have been utilized to enhance the performance of LSPR sensors, including various geometrical and material parameters, plasmonic wave coupling from different structures, and integration of noble metals with graphene, which is the focus of this report. Recent studies of metal-graphene hybrid plasmonic systems have shown its capability of promoting the performance of the LSPR sensor to a level that enhances its chance for commercialization. In this review, fundamental physics, the operation principle, and performance assessment of the LSPR sensor are presented followed by a discussion of plasmonic materials and a summary of methods used to optimize the sensor’s performance. A focused review on metal-graphene hybrid nanostructure and a discussion of its role in promoting the performance of the LSPR sensor follow.

## 1. Introduction

The Surface Plasmonic Resonance (SPR) sensor is a common and commercialized sensor used in different areas such healthcare and gas sensing [1,2]. When SP evanescent waves and incident light couple together, Surface Plasmon Polaritons (SPP) result, which is the basic element in SPR sensor principles. Any variation in permittivity of a sensing area shifts the resonance angle of the SPP and the resonant intensity [3,4]. When the size of the plasmonic material decreases to the nano range, the way in which free electrons couple with the incident wave differs. When metallic nanoparticles are irradiated by an electromagnetic wave with a wavelength greater than the NP size, the metal’s free electrons generate oscillations called Localized Surface Plasmons (LSPs). These collective oscillations are restricted to the geometry of the nanostructure [5]. LSPs can be induced by direct illumination of photons where there is no need for momentum matching between a photon and free electron [5]. The only required condition to induce LSPs is conservation of the electron energy of the metal nanoparticle (NP) with incident photon energy.

In addition to the low cost of fabrication, the LSPR sensor offers a simple sensor setup and detection of local change in the refractive index, which is not the case with SPR sensors [6,7]. However, damping in the radiation process in LSPR modes increases the resonance peak width, and accordingly reduces sensor performance [8,9,10]. Different approaches have been reported to enhance LSPR sensor performance, including variation of the geometry and material type of the nanostructure, surface plasmon hybridization, and noble metal-2D material integrated structures such as metal-graphene hybrid nanostructures. However, recently, metal-2D material integrated structures showed good improvement in LSPR sensor performance, the focus point of this review paper. This review starts by summarizing the fundamentals of the localized surface plasmon resonance sensor, followed by a discussion of the material used in the plasmonic sensor. A summary of the different approaches used to enhance the performance of the LSPR sensor follows, with additional focus on a metal-graphene material integration approach.

## 2. Localized Surface Plasmon Resonance Sensor

The Mie scattering theory, with the help of Maxwell’s equations, is a very useful way to explain how metal NPs interact with incident light. Equation (1) shows the derived optical extinction for metallic NP with a radius smaller than the wavelength of the incident wave (2r << λ) [5]:

(1) $$\sigma_{ext}\left(\lambda \right)=\frac{24\pi^{2}r^{3}\varepsilon_{d}^{3/2}N}{\lambda \ln\left(10\right)}\frac{\varepsilon_{i}\left(\lambda \right)}{{\left(\varepsilon_{r}\left(\lambda \right)+2\varepsilon_{d}\right)}^{2}+\varepsilon_{i}{\left(\lambda \right)}^{2}}$$

where *N*, λ, $\varepsilon_{d}$, $\varepsilon_{r}$, $\varepsilon_{i}$ are the electron density, incident wavelength of the incident wave, permittivity of the surrounding medium, and the real and imaginary part of the metal NP’s permittivities, respectively. The metal NP behaves as an electric dipole under a uniform static electric field assumption, and its polarizability, α(λ), is derived as:

(2) $$\alpha \left(\lambda \right)=4\pi r^{3}\frac{\varepsilon \left(\lambda \right)-\varepsilon_{d}\left(\lambda \right)}{\varepsilon \left(\lambda \right)+2\varepsilon_{d}\left(\lambda \right)}$$

**Figure 1:**
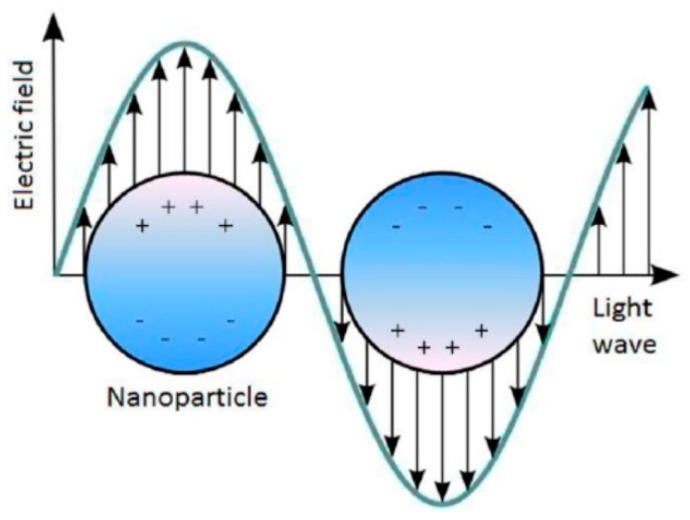
Metal NP external electric field interaction [17].

When $\varepsilon \left(\lambda \right)\approx -2\varepsilon_{d}\left(\lambda \right)$, the dominator of Equation (2) approaches zero and yields maximum polarization: in order to achieve this condition, the dielectric function (real part) ε(λ) of the NP requires a negative value, where the dielectric function of the surrounding medium is considered as a constant, and the losses (imaginary part) of the NP are assumed to be small [11]. Using the Drude model, the resonance frequency ($\omega_{LSP}$) of a dipolar LSP can be given by [12]:

(3) $$\omega_{LSP}=\frac{\omega_{p}}{\sqrt{1+\varepsilon_{d}}}$$

where $\omega_{p}$ is the plasmon frequency of the NP’s material. At the resonance frequency, as shown in Figure 1, the free electrons moved away from their equilibrium position, forming an electric dipole structure. After excitation, the electrons are displaced from their atomic positive core and create an attraction columbic force that works as a restoring force. As a result, collective oscillations are created and decay within a few femtoseconds [13]. Excitation with the electric field produces a continuous plasmon oscillation [13]. Furthermore, the field is enhanced in the proximity of the NP at plasmon extinction, which has been utilized in different plasmonic applications such as an LSPR sensor [14,15,16].

Mie resonance is a good example of the nonlinear optical effect where the optical response of the medium is nonlinearly depending on the electric field applied [16]. In addition to the applied electric field, there are a number of parameters that affect the plasmonic resonance properties. When the surrounding’s dielectric constant increases, screening of the surface electrons increases. This results in the increase of the restoring force and, hence, requires lower energy to excite free charges. The resonance peak consequently shifts to the red region [14], as can be seen from Equation (3). From this equation, it is also evident that the increase in the surrounding’s permittivity or refractive index reduces the resonance frequency and hence results in a longer resonance wavelength shift. Therefore, metallic NPs can be used as an LSPR sensor where both the polarization of the NP and restoring force strength affect the sensitivity of the sensor; accordingly, sensor performance depends on the type of materials and the geometrical properties of the metal NPs. According to the equation mentioned above, as the NP size increases, the resonance wavelength shifts to a longer wavelength, which is a kind of tuning of the plasmonic resonance [18]. In addition, increasing the size of particles increases the radiative losses, which increases the width of the resonance band, hence resulting in intensity reduction [19]. Furthermore, NP geometry has a crucial role in tuning the resonance wavelength. The NP shape applies an important impact effect on the plasmon resonance peak position. In the case of a high symmetry spherical NP, only one dipolar resonance is induced. However, when the shape is altered, the particle becomes increasingly asymmetric, and a higher order of electric dipole modes is induced, which increases the complexity of optical response of the NP [20]. For example, upon exciting a nanorod, two distinct resonance peaks are observed, one from the transverse and one from the longitudinal mode at different spectral positions [14]. Furthermore, resonance with higher order modes, for example, quadrupoles and octopoles, may be induced in the case of an inhomogeneous distribution of the surface charges of the NPs [21,22,23]. Therefore, engineering the shape of the NP is an effective way to generate LSP resonance modes within a visible and near-infrared (NIR) region [24,25].

Nanostructure supported LSPR extinction can be used in refractometric sensing, like a conventional SPR sensor. The detection principle in LSPR sensors is based on an intensity or wavelength shift upon variation in the permittivity of a sensing region (proximity region of the NP). In the transmission or reflection spectrum, the resonance peak position varies based on the refractive index of the sensing area, as is confirmed by Equation (3). Any change of permittivity in the proximity of the NP will shift the resonance LSP peak, which can be employed to detect molecular interaction between the NP and adsorbed material. The amount of adsorbed material can be found through monitoring the plasmonic resonance shift. The performance of the sensor can be assessed through Sensitivity (S) and Figure of Merit (FOM) measurements. Sensitivity (S) is defined as the extent of peak shift in position or intensity to the change of the refractive index of a dielectric medium. Therefore, a larger resonance shift at a small refractive index change results in increased bulk sensitivity [26]. It is known that increased sensitivity due to larger surface plasmon resonance wavelength shifts results in a wider resonance peak, due to dephasing and radiative damping, which affect the resolution of detection [8]. Therefore, sensitivity measurement is not adequate to assess the performance of the sensor. Thus, another parameter required to assess the LSPR sensors is called the Figure of Merit (FOM). FOM is the sensitivity divided by bandwidth (Full Width at Half Maximum (FWHM)) in the case of a wavelength shift, or by reference intensity in the case of an intensity shift. Equations (4)–(7) are the sensitivity and FOM equations for both the wavelength shift and intensity shift [8].

(4) $$S_{\lambda }=\frac{\Delta \lambda }{\Delta n}\;\left(nm/RIU\right)$$

(5) $$S_{I}=\frac{\Delta I}{\Delta n}\;\left(RIU^{-1}\right)$$

(6) $$FOM_{\lambda }=\frac{S_{\lambda }}{\mathrm{FWHM}}$$

(7) $$FOM_{I}=\frac{S_{I}}{I_{ref.}}$$

where Δλ is the change that occurs in the resonance wavelength (nm), ΔI is the change in intensity, Δn is the change in the sensing medium’s refractive index, and $S_{\lambda }$, $FOM_{\lambda }$, $S_{I}$, $FOM_{I}$ are the sensitivity and figure of merit based on the wavelength and intensity shift, respectively. Therefore, a higher resonance shift is preferred at a small refractive index change to achieve higher bulk sensitivity and FOM. Thus, both a large Δλ and narrower resonance peak is desired to promote the quality of an LSPR-based sensor.

## 3. Plasmonic Material

Metal is a candidate material for a plasmonic device. It has an enormous number of free electrons, and upon excitation with electromagnetic wave, those free electrons produce collective oscillations, which is the plasmon phenomena used in optical devices. Any plasmonic material should have the ability to provide a negative real permittivity to be used in a plasmonic device. Free electrons in metal provide this property, and metal therefore is mostly used as a plasmonic material [11]. However, due to interband electronic transition in metals, they suffer large losses in the Visible (Vis) and Ultra-Violet (UV) frequencies [27]. These losses affect the performance of plasmonic devices and limit their applications. At optical frequencies, silver has minimum loss compared to other metals, and thus, it is the best choice for a plasmonic device. At around a 500-nm wavelength, gold has larger loss than silver; however, it is mostly used as a plasmonic material at a lower NIR region due to its chemical stability [11]. Another example of a metal that is used as a plasmonic material is copper, which has higher losses than silver and gold for most optical wavelengths. One of the applications of plasmonic devices is LSPR sensors, in which gold is mostly used as the plasmonic material due to its chemical stability [28]. It has been reported that some metals with higher optical losses than gold and silver have been used as plasmonic materials when functionality affects the overall performance of the device, such as platinum and palladium, where these were used as catalytic materials in addition to their use as plasmonic materials [29,30]. Different review papers give additional details about the use of metal as a plasmonic material [7,31,32].

Graphene, a single layer of carbon, has been reported as a new emerging competitive plasmonic material to metal. In the NIR-IR region, graphene has low losses and high confinement of surface plasmons and, thus, has a good advantage over metals [33]. Furthermore, graphene can support SPs in a flexible and curved structure. When combining graphene with metal, the tunability of a plasmonic device is enhanced. As stated, metal has losses in the visible spectra region, and losses increase at lower frequency, which is the opposite case to graphene [33]. Therefore, a graphene-metal hybrid nanostructure increases the tunability of plasmonic devices in a wide range of frequency, starting from visible down to the infrared region. Different reviews about plasmonics in graphene and its application have been reported in the literature [33,34], which give good details about plasmonic physics and applications focusing on graphene itself. However, in this review, the focus is on a metal-graphene hybrid nanostructure and the effect of the integration of metal with graphene on plasmonic sensor performance.

## 4. Different Approaches Used to Enhance LSPR Sensor Performance

The performance of plasmonic sensors is directly related to the amount of energy loss in the metallic structure of a sensor. For example, using a metallic structure with a high loss results in broadening of the resonance plasmonic waveband, and hence, the FOM of a sensor is decreased. It is known that silver has a lower damping rate and thus has a lower absorption loss than gold and copper [27]. Gold and copper are still considered to be low-loss materials, close to silver, compared with aluminum, which has the highest losses in the visible spectrum [27,35,36]. A plasmonic sensor, e.g., an LSPR sensor, requires a material that has lower losses to achieve better refractive index sensitivity. When comparing gold and silver, silver offers better sensitivity than gold [37,38]. Upon using a spherical NP with a diameter of 60 nm, silver exhibits better sensitivity (160 nm/RIU) (i.e. RIU: refractive index unit) compared to that of gold (60 nm/RIU). The main reason for observing a higher refractive index sensitivity in silver is attributed to a lower loss in silver and, hence, a lower damping rate, which results in increasing scattering efficiency and therefore refractive index sensitivity [38]. However, losses of material are not the only physical property that needs to be considered in the design of an LSPR sensor. The compatibility of the sensor with the sensing medium is also essential. If the material used in an LSPR sensor is corrosive in an aqueous medium or oxidized in air, refractive index sensitivity diminishes significantly [39]. Therefore, since gold has higher absorption loss than silver, it is preferred in most LSPR applications. Silver [40] and other metals such as copper [41], aluminum [42], or metal oxides such as zinc oxide [43] are corrosive in aqueous medium and oxidize in air, which limits their application in LSPR sensor engineering. In addition to the material type of the NP used in the LSPR sensors, NP size also affects the refractive index sensitivity of sensors. Particle size affects the position of the surface plasmon peak, and it shifts towards larger wavelengths upon increasing the size of the NPs, thus enhancing the sensitivity where it (i.e., sensitivity) is higher at larger wavelength resonances. It has been verified that the SP peak of Au NPs can be redshifted up to 60 nm upon increasing the particle radius from 10–100 nm [44]. This gives a good ability to engineer plasmonic resonance by tuning the wavelength of the SP resonance of NPs for different applications. Therefore, the proper choice of NP size must be considered to achieve better sensitivity of a plasmonic sensor.

The shape of the NP is also used as a tunable parameter to improve LSPR sensor performance. Changing the shape of NP causes resonance peak shift [17]. Tuning particle shape is a method used to tune the wavelength of resonance at longer wavelengths and, hence, optimize the sensitivity of an LSPR sensor. Mock et al. studied the effect of different shapes (sphere, cube, and triangle) of silver NPs on the sensitivity of a single NP to the change in the refractive index of the surrounding medium [38]. They reported that the triangle NP exhibited larger sensitivity (i.e., 350 nm/RIU) than a spherical NP (i.e., 160 nm/RIU). Sun et.al [45] showed that a gold nanoshell shows higher sensitivity (i.e., 406 nm/RIU) than a gold spherical NP with a sensitivity of 60 nm/RIU. The main reason for observing higher sensitivity in the triangle [46], nanoshell [45], and nanocube [8] structures relative to the spherical NPs [4] is due to the sharper edges of these NPs, which increase the intensity of the localized electric field around the NPs. Changing the aspect ratio also helps to produce multi-modal resonances at longer wavelengths, thus increasing sensor sensitivity. Hanarp et al. studied nanodisc structures with aspect ratios starting from 1:1–5:1 at refractive indices change from 1.30–1.50 [47]. More resonance shift was observed in the case of the 5:1 than in the case of the 1:1 aspect ratio, which offers better sensor performance. Enhancement in sensitivity is acquired due to strongest excitation resulting from the elongated nanodisc resonance mode [47]. Thus, the aspect ratio is another powerful key used to tune the plasmonic properties of a nanostructure.

By using two or more NPs separated by a specific distance in one-, two-, or three-dimensions, the interaction between these incident waves and the excited surface plasmon wave from NPs resulted in an additional shift in the resonance wavelength to the red region as a result of the coupling interaction, which enhances sensor sensitivity [48]. Erik et al. [49] studied the effect of inter-coupling between two gold NPs (10 nm in diameter) on electric field enhancement. NPs’ spacing affects the position of resonance peak and decreases the separation distance; the resonance wavelength shifts toward a longer wavelength. However, upon using a structure with a larger separation distance between the NPs than the NPs’ dimensions, the resonance wavelength shift is negligible, as discussed elsewhere [50]. Furthermore, upon reducing the distance between two consecutive NPs, the electric field in a near-field regime is enhanced in the gap between NPs’ “hot spot”. Erik et al. also showed that the induced electric field distribution between two consecutive NPs with a separation distance of 3 nm is stronger than in the case of 10 nm. As mentioned earlier in this report, due to the nonlinear effect, the number of parameters affects the plasmonic properties, such as field strength. In Erik et al.’s report, it was shown that keeping NP’s geometry and material fixed, a lower inter-particle distance is preferred to enhance the local field. Increasing the amount of scattering results in enhancement of the sensitivity of the LSPR sensor [14,15].

Plasmon mode hybridization is another promising method that has been applied to promote the performance of LSPR sensors. Nanorings and nanoshells are the best nanostructures that represents the plasmon hybridization effects [51]. In general, two different plasmonic modes are observed from the hybridization phenomena: bonding and antibonding modes [52]. The bonding mode and anti-bonding mode, which are observed at a lower and a higher frequency, respectively, result from the symmetrical and asymmetrical coupling of induced plasmonic waves and incident light in a nanostructure [52]. The geometry of the nanostructure plays a crucial rule in tuning hybridized plasmonic modes. Tao et al. [53] showed that by using nanoring structures, larger sensitivity could be achieved than with nanodisc structures. Sensitivity improvement is attributed to strong localization of the field in the ring’s center. They also reported that the effect of ring width variation is more significant for the spectral properties than other geometrical parameters, such as the ring size and the rings’ separation distance. As ring width is increased, the plasmonic resonance peak narrows, which helps to enhance sensor performance and, specifically, the FOM of the sensor due to the reduction of the FWHM of the resonance peak. There are several other studies on nanostructures and the plasmonic hybridization effect, such as hollow nanoshells [45], nanorice shells [54], and nanotube arrays [55], and using nanoparticle arrays; coupling plasmonic waves and incident waves shows an improvement in plasmonic resonance properties, and this could improve LSPR sensor performance [45,56].

## 5. Metal-Graphene Hybrid LSPR Sensor

**Figure 2:**
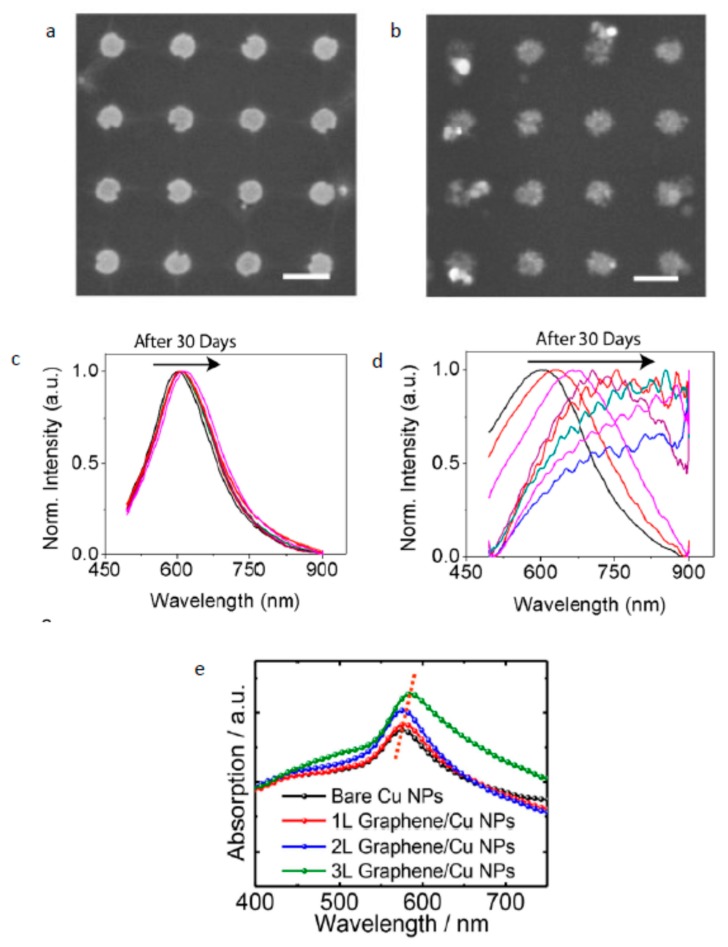
Scanning electron microscopy (SEM) images of a bare Ag nanoantenna (**a**) and a graphene-passivated Ag nanoantenna (**b**) after 30 days (scale bars are 200 nm), showing how graphene protects Ag NPs from degradation [60]. Bare Ag nanoantenna’s (**c**) and graphene-passivated Ag NPs’ (**d**) normalized (Norm.) reflection spectra over 30 days, which shows how the passivation of Ag with graphene enhances the stability of Ag NPs [60]. Optical absorption spectra for the Cu NPs coated with a few graphene layers [61].

Longer wavelength resonance, high resonance intensity or amplitude, and sharper resonance band (min FWHM) are indications of enhanced sensitivity and FOM of the LSPR sensor. Therefore, engineering a plasmonic material by choosing a proper type, tuning the geometry of the NP, or hybridizing it with another material in a way that improves one of the mentioned three plasmonic properties could lead to promoting plasmonic sensor performance. Starting with the type of plasmonic material, silver is the best choice among other plasmonic materials in terms of sensor performance due to low optical loss compared to other metals in the visible range. This is due to the lack of the interband transition as in gold and copper [11]. However, in addition to the oxidation problem, Ag_2_S forms on the Ag surface due to the reaction of carbonyl sulfide (OCS) and hydrogen sulfide (H_2_S) with the Ag surface; this affects the plasmonic properties of the Ag nanostructure and results in an increase in material loss [57] Furthermore, another source of loss arises from scattering due to the increase in the surface roughness of the Ag surface [58]. Gold for this reason is used more in applications even though gold suffers from optical loss in the visible range. Passivating and encapsulating of the Ag surface to prevent the formation of ${\mathrm{Ag}}_{2}S$ and maintaining the excellent plasmonic properties of Ag nanostructure have been reported [59]. However, the thickness of the passivation layer is high, up to a level that affects near-field interaction, which is very strong in the vicinity of the nanoparticle [59,60]. Therefore, a passivation material that prevents the formation of ${\mathrm{Ag}}_{2}S$ and that is sufficiently thin is desired to help solve the limitation of Ag in LSPR sensor applications. Leenaerts et al. studied the permeability of a graphene sheet with different levels of defects for helium gas molecules [60]. They found that the penetration of the defective graphene sheet for small atoms and molecules decreases exponentially with the decrease in the size of sheet defects, and thus, a large defect size is preferred to increase graphene permeability for gas molecules. This result led to the use of graphene as a passivation layer for Ag NPs, in addition to the good plasmonic properties of graphene itself. Jason et al. studied the effect of graphene passivation on the plasmonic properties of Ag NPs. They fabricated two sets of a Ag nanoantenna array, and one of these was passivated with graphene by transferring CVD-grown graphene on the Ag nanoantenna array, as shown in Figure 2a,b. From the SEM image of the passivated and unpassivated array, it is clear how ${\mathrm{Ag}}_{2}S$ formed on the Ag surface affects the morphology of the Ag nanoantenna, which affects plasmonic resonance over 30 days of measuring optical reflection. They observed that the resonance peak shifts 216 nm after 30 days of fabrication for the unpassivated arrays, while a very large reduction in resonance shift of 15 nm occurs in the case of graphene-passivated Ag NPs; see Figure 2c,d. This provides high stability of the plasmonic properties of the Ag NPs over the time it is passivated with graphene. To assess the effect of passivating Ag NPs with graphene on LSPR sensor performance, they measured the sensitivity of passivated a Ag NP array and a Au NP array by monitoring the resonance shift over change in the refractive index of the surrounding medium. The sensor sensitivity of the Au NP array was found to be 102 nm/RIU and jumped to 162 nm/RIU for the graphene-passivated Ag NP array, which represent ~60% enhancement in the sensor performance. This enhancement in performance of an Ag-based sensor after using graphene as a passivation material means that graphene is a promising material to solve the problem of utilizing an Ag-based LSPR sensor in different applications. Another example showing the effectiveness of using graphene as a passivation material for an LSPR device is with passivating copper NPs, as reported by Li et al. [61]. Copper is a good plasmonic material after silver and gold due to it having higher loss, but its low cost compared to gold and silver gives it a good advantage. However, under an ambient environment, copper oxidized rapidly with degradation in the LSPR intensity resulting and, thus, a reduction in the sensing performance [62,63]. In order to overcome this issue, protecting the Cu surface from oxidization is required. As mentioned, protecting the NPs with a thin layer of material helps to detect the near-field effect of plasmonic NPs after being excited with light. Li et al. transferred CVD graphene (1–3) layers on top of a Cu NP array fabricated on a quartz substrate, and the absorption spectra were measured; see Figure 2e. They observed drastic field enhancement after adding graphene, and adding more graphene layers enhanced the intensity of plasmonic resonance. This enhancement is due to strong field localization at the graphene/copper interface that resulted from the transfer of electrons from graphene to the surface of Cu due to the higher work function of Cu compared to graphene, as reported in [64]. Furthermore, the resonance peak redshifted with increasing graphene layers resulting from an increase in the refractive index of the Cu surrounding medium. Both enhancements of plasmonic resonance intensity and redshift in the position are indicators of the enhancement of the performance of this device when used as an LSPR sensor. Therefore, in addition to protecting the Cu surface from oxidation, graphene by its plasmonic properties also enhances the plasmonic properties of a sensor-based device.

Decorating graphene film with metal NPs is another approach used to tune the plasmonic properties of an LSPR device. Xu et al. fabricated Ag NPs on top of a CVD graphene film using a thermally-assisted self-assembly method [65]. They observed that as the Ag NP size increased from 50 nm–150 nm, plasmonic resonance redshifted from 446–495 nm. This redshift can be attributed to two reasons. The first is the increase in the size of Ag NPs, for which is known that as the metal NP size increases, longer wavelength resonance is produced [44]. Furthermore, Xu et al. reported that as the graphene film thickness increases, plasmonic resonance redshift and amplitude are reduced. Graphene is a good conductor, and thus, it facilitates energy concentrated in the Ag NP to transfer to the graphene film, consequently decreasing the amplitude of plasmonic resonance [65,66]. Red shifting of resonance is a good behavior to enhance the sensitivity of the LSPR sensor, as mentioned; however, decreasing the amplitude of the resonance results in an increase in the FWHM of the resonance band, thus a reduction in the FOM of the sensor as explained in Equation 6. Therefore, during the design of a metal-graphene hybrid LSPR sensor, the size of metal NP and graphene has to be done in manner that gives longer wavelength resonance with a high amplitude or minimum FWHM.

**Figure 3:**
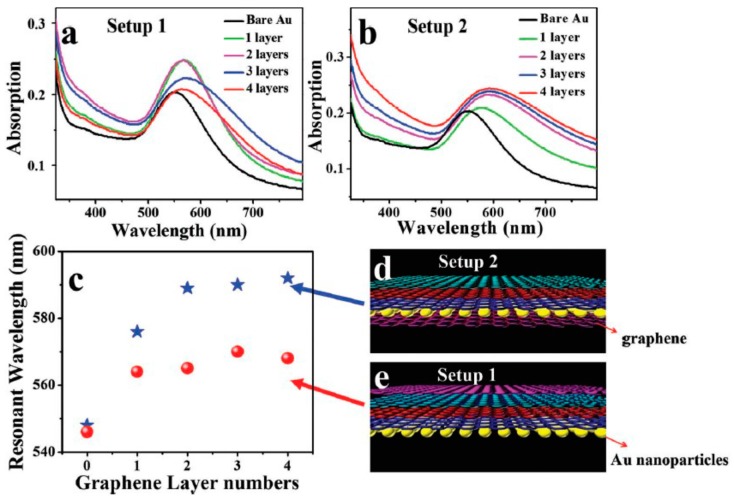
Absorption spectra of gold NPs coated (**a**) and encapsulated (**b**) by graphene layers. (**c**) Gold NP LSPR wavelengths for Setups 1 and 2. (**d**,**e**) The structures of the setups used in (**b**,**a**) [67].

Another metal-graphene hybrid system that studied the effect of the hybrid nanostructure on the plasmonic resonance of the nanostructure is that reported by Nan et al. [67]. They studied the effect of two different gold-graphene hybrid systems on the plasmonic resonance of a nanostructure. The first hybrid structure covered Au NPs with graphene (Figure 3e), and the second one was “sandwiched” Au NPs with graphene layers (Figure 3d). For both hybrid schemes, there is an obvious redshift for the resonance peak (Figure 3a,b), but there is no clear effect on red shifting as graphene layers increase (Figure 3c). The redshift in plasmonic resonance is attributed to electron transfer from Au NPs to graphene. Consequently, modulating the electron transfer process could provide the ability to tune plasmonic resonance and, thus, improve sensor performance by producing longer wavelength resonance.

A plasmonic material that has high absorption in the visible region, such as metal, is preferred in plasmonic applications such as an LSPR sensor. Graphene has very low absorption in the visible spectrum, and thus, enhancing its absorption will increase its chance to be used as a plasmonic material for an LSPR sensor in the visible region [68]. The hybrid of graphene with metal enhances graphene absorption, as reported by Wu et al. [69]. They fabricated graphene nano mesh samples and filled the empty part of the mesh with Au nanodiscs. They observed a huge improvement in the absorption of the hybrid nanostructure compared to bare graphene. This improvement can help to enhance the LSPR-based sensor from two sides. First, enhancing absorption will increase the localized field around the nanostructure, and thus, it will be more sensitive to a change in the surrounding refractive index. Furthermore, the increase in the absorption band could lead to a decrease in the FWHM of the resonance band, thus enhancing the resulting FOM of the sensor. Therefore, integration of graphene with metal increases the opportunity for a graphene-based device to be used in the visible region for different LSPR sensor applications.

Increasing the metal NP size results in red shifting in plasmonic resonance; for this reason, having a fabrication method that can control the size of the metal NPs on top of graphene film is important. Lee et al. introduced a tunable size and LSP resonance of Au NPs on top of a mono-layer of graphene film based on the reduction potential between the Au^3+^ precursor and graphene [70]. They modulated the Au NP size by the concentration of Au^3+^ precursor, the surface energy of graphene on the substrate, the spin-coating speed, and the coating cycles. However, they found that Au NP size is strongly affected by the concentration of Au^3+^ precursor and the coating cycle’s number. Upon increasing the concentration of Au^3+^ and repeating the cycles of the reduction process, the plasmonic resonance of the Au-graphene hybrid nanostructure was redshifted, and broadening in the band FWHM was observed. After repeating the reduction process, the Au NP size was distributed between 20 and 120 nm, and the resonance redshifted from 560–620 nm. This redshift in resonance wavelength is a tuning parameter to enhance LSPR sensor performance; however, as mentioned in the previous section, the FWHM of the resonance band has to be minimized to obtain a superior FOM of the sensor.

**Figure 4:**
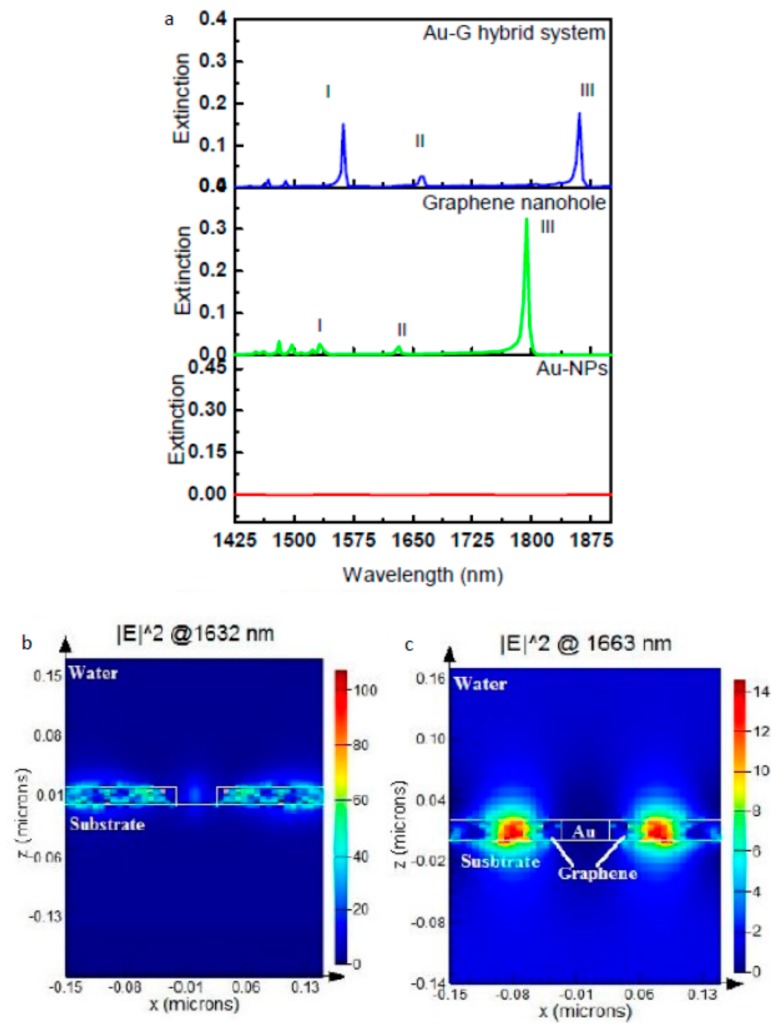
(**a**) Extinction spectrum for three different nanostructures; Au NP square array, nanohole array perforated in 20 nm-thick graphene film, and Au NPs/G hybrid structure showing three main resonance modes; Mode I (λ = 1532 nm), Mode II (λ = 1632 nm), and Mode III (λ = 1793 nm). (**b**,**c**) are the electric field profiles at the resonance wavelength of Mode II for the graphene nanohole structure and the Au-graphene hybrid structure, respectively [24].

Recently, some published works showed the direct effect of hybrid graphene with metal on LSPR sensor performance. Maurer et al. studied the effect of the graphene layer as a spacer between Au film and Au NPs. They deposited graphene film on top of a gold film and then decorated the layer with an Au nanodisc array [28]. They compared the optical extinction between AuNPs/Au film and AuNPs/graphene film/Au film nanostructures. Enhancement in the localization field after adding the graphene spacer led to an ~33% enhancement in the sensitivity of the sensor. Furthermore, a sharper resonance width resulting from the Au-graphene hybrid structure was achieved and increased the FOM from 2.1 for Au NPs/Au film to 2.8 for Au NPs/graphene/Au film nanostructures. This work done by Maurer gives evidence of the effectiveness of the metal-graphene hybrid nanostructure on the performance of the LSPR sensor. In one of our published works [25], the plasmonic properties of the Au-graphene core-shell NP array on top of a quartz substrate have been theoretically investigated using a Finite Difference Time Domain (FDTD) method. By comparing the extinction of the Au NP array with the Au-graphene core-shell NP array, very strong enhancement was present in the resonance band in the NIR region, in which sharpness reached an FWHM of 7.8 nm. This sharp resonance resulted from a strong localization field enhancement after coating the Au NPs with a graphene shell due to electron transfer between the Au NP and graphene shell, as shown clearly in electric field profiles. Furthermore, the periodic arrangement of the hybrid NPs enhanced the interaction field between them and enhanced the resonance, which is clear by the increase in the resonance amplitude when the periodicity (i.e., distance between NPs) decreased. The FOM of this Au-graphene core-shell NPs array nanostructure has been tested by measuring the optical transmission at a different refractive index value (Δn = 0.01) of the surrounding medium. The results showed a huge improvement in the FOM compared to the related published work in the field of metal-graphene hybrid LSPR sensors [28], by achieving an FOM of 102.6. Furthermore, this improvement in the NIR region could be attributed to the low loss of graphene compared to metal. Another hybrid system studied in our group is a planner Au-graphene hybrid LSPR sensor [24]. In this hybrid nanostructure, the plasmonic properties of the Au NP array/graphene film hybrid scheme have also been theoretically studied using an FDTD method. The graphene in this nanostructure fills the gap between the Au NPs array, which shows a huge improvement in the extinction in the NIR region (Figure 4), as reported in the previous published work [25]. This indicates that the NIR region is good for the hybrid of graphene with metal. It was observed that the intensity of the plasmonic resonance of this hybrid system is extremely sensitive to very small changes in refractive index value (Δn = 0.001) and results in an FOM of 390 and a sensitivity of 4380 nm/RIU. This improvement in the FOM of the LSPR sensor opens the door for the competition of a commercialized SPR sensor and promotes the commercialization opportunity of an LSPR sensor.

## 6. Conclusions

In this short review, the fundamental physics of the LSPR sensor was summarized followed by a discussion of different methods used to enhance the performance of LSPR sensors. It was shown that the size and shape of the nanoparticle give a broad tunability in optimizing the performance (sensitivity and FOM) of the LSPR sensor. A smaller NP size (<100 nm) is preferred for better plasmonic properties, as reported in [25]; however, the fabrication ability should be carefully considered to assure reliability and reproducibility. Furthermore, NPs with sharp edges give better performance, but nanodiscs from the fabrication side are more feasible and show good sensing performance [24]. Furthermore, choosing proper material is a crucial factor affecting device performance. It seems that tuning the plasmonic properties of an LSPR device via geometrical parameters still needs more work to produce a competitive result in term of sensitivity and FOM. However, a metal-graphene hybrid nanostructure has so far shown huge promotion in enhancing the performance of the sensor. Recently, integrating metal-graphene material plasmonic devices using a core-shell hybrid nanostructure has been offering significant improvement in LSPR sensor performance with an FOM of 102.6 [25]. More enhancement in sensitivity (4380 nm/RIU) and FOM (390) were achieved using the Au NP/graphene film hybrid nanostructure, with a very small change in the refractive index (Δn = 0.001) [24]. As reported in [71], a plasmonic sensor with a sensitivity of 1015 nm/RIU has a 15-ng/mL detection limit. Thus, a sensor with a four-order higher sensitivity at a very low refractive index change (Δn = 0.001) [24] could achieve a much lower detection limit, which may reach down to atto g/mL (ag/mL). A plasmonic sensor with the ability to detect very low change in analyte concentration would be a game-changer in molecular sensing, especially in early-stage disease diagnosis.
